# Maternal celiac disease autoantibodies bind directly to syncytiotrophoblast and inhibit placental tissue transglutaminase activity

**DOI:** 10.1186/1477-7827-7-16

**Published:** 2009-02-19

**Authors:** Naheed Anjum, Philip N Baker, Nicola J Robinson, John D Aplin

**Affiliations:** 1 Maternal and Fetal Health Research Group, University of Manchester, Research Floor, St Mary's Hospital, Manchester, M13 0JH, UK

## Abstract

**Background:**

Celiac disease (CD) occurs in as many as 1 in 80 pregnant women and is associated with poor pregnancy outcome, but it is not known if this is an effect on maternal nutrient absorption or, alternatively, if the placenta is an autoimmune target. The major autoantigen, tissue transglutaminase (tTG), has previously been shown to be present in the maternal-facing syncytiotrophoblast plasma membrane of the placenta.

**Methods:**

ELISA was used to demonstrate the presence of antibodies to tissue transglutaminase in a panel of CD sera. Immunohistochemistry was used to evaluate the binding of IgA autoantibodies from CD serum to term placenta. In addition, novel direct binding and activity assays were developed to mimic the in vivo exposure of the villous placenta to maternal autoantibody.

**Results and Discussion:**

CD IgA autoantibodies located to the syncytial surface of the placenta significantly more than IgA antibodies in control sera (P < 0.0001). The distribution of antigen was similar to that observed using a monoclonal antibody to tissue transglutaminase. Staining was reduced by pre-absorption of CD serum with recombinant human tissue transglutaminase. In direct binding assays, autoimmune immunoglobulin A (IgA) from the maternal compartment became associated with antigen at the syncytial surface of the placenta, as a result of which transglutaminase activity at this site was inhibited.

**Conclusion:**

These data indicate that direct immune effects in untreated CD women may compromise placental function.

## Background

Celiac disease (CD) is caused by intolerance to dietary gluten, resulting in immunologically-mediated inflammatory damage of the small intestinal mucosa, malabsorption and nutritional deficiency[1,2]. The enzyme tissue transglutaminase (tTG) has been identified as the major autoantigen in CD[3]. tTG is a multifunctional protein that catalyses the formation of cross-links between proteins, has GTPase activity associated with G-protein-linked signalling[4] as well as being a kinase[5]. tTG is widely expressed in tissues, where it is often found associated with cell membranes[1,6]. Its functions appear to be diverse: one important hypothesis that is supported by *in vivo *data suggests that tTG is important in the regulation of late events in apoptosis, when cellular remnants are stabilized by cross-linking in preparation for disposal in the absence of inflammatory stimuli[7].

With the wide availability of sensitive serological screening tests that detect anti-endomysial (EMA) and anti-tTG antibodies, it has become apparent that the prevalence of CD is higher than had been previously suspected [8-11]. Many, if not most, cases have either a clinically silent form of the disease, or only a minor enteropathy[12,13]. Untreated celiac disease has been associated with poor pregnancy outcomes including higher rates of infertility, recurrent miscarriage, intrauterine growth restriction (IUGR), and stillbirth [14-20]. IUGR, perhaps the most predictable potential outcome of impaired maternal nutrient absorption, is an important cause of perinatal morbidity and mortality as well as giving rise to increased risk of poor health in adult life[21,22]. A 9-fold increased incidence of IUGR has been reported in CD[23] with equivalent effects in women with subclinical disease and an estimated 1 in 80 pregnancies may be affected by CD. This incidence is comparable to the incidence of diabetes [24] and thyroid disease [25]. Studies to characterize CD pregnancies are constrained by the likelihood that transferring affected women to a gluten-free diet (GFD) would improve outcome. Therefore, data on placental development and fetal growth in CD are scant and the mechanisms by which pregnancy may be affected are not established.

In order to develop an evidence base from which to judge whether routine CD screening should be instituted in pregnant women, there is a pressing need for *in vitro *approaches to understand mechanisms of pregnancy impairment. A central question is whether maternal malabsorption may be complicated by direct immune attack on the placenta. Functionally active tTG is present at the syncytiotrophoblast microvillous membrane (MVM) [26-28], where a group of substrate proteins has been identified[28]. The MVM is the primary exchange interface between maternal and fetal tissues and is perfused directly by maternal blood. We have suggested a role for tTG in trophoblast apoptosis and shedding from this surface[28]. In the present study we use novel binding and function assays to show that CD-derived IgA binds tTG at the maternal surface of the placenta and inhibits its function. The results suggest that CD placentas may carry a high autoimmune immunoglobulin load, leading to developmental or functional impairment.

## Methods

### Serum and EMA assay

Anti-endomysium antibodies (EMA) were determined by indirect immunofluorescence on pig intestine. 132 serum samples from non-pregnant donors were provided by the immunology laboratory of the Manchester Royal Infirmary and stored at -20°C. EMA-positive sera were reassayed blind at 1:30, 1:100, 1:300 and 1:1000.

### tTG assay

A commercial ELISA (Celikey; Pharmacia Diagnostics) was used to determine anti-tTG IgA levels in patient sera. Results are reported as positive (OD ratio >1.4), borderline (OoralD ratio 1–1.4) and negative (OD ratio <1). The presence of tTG reactive IgA was confirmed by western blotting (not shown).

### Immunohistochemistry

Sections of normal term placenta were dewaxed and incubated in methanol containing 0.15% hydrogen peroxide for 30 min to quench endogenous peroxidase activity, then microwaved in 0.01 M sodium citrate buffer pH 6.0, 10 min to achieve antigen recovery. Sections were incubated with protein block for 30 min then with autoimmune serum (tTG-positive or -negative; 1:10) overnight at 4°C. Mouse monoclonal anti-tTG (CUB 7402; 1:100, Labvision) was used as a positive control[26].

Sections were rinsed in TBS (x3) then incubated with either goat anti-mouse immunoglobulin-HRP or rabbit anti-human IgA-HRP (1:40, 30 min; Dako P0447 and P0216 respectively). After washing, sections were incubated with avidin-peroxidase (5 μg/ml in 0.125 M TBS, 1 h), developed in 3, 3' diaminobenzidine tetrahydrochloride (DAB) (10 mg/ml DAB/TBS, 0.0045% hydrogen peroxide) for 3 min, then nuclei counterstained with 0.25% methyl green.

The stained area (for syncytioplasm) or the linear staining (for the syncytial microvillous membrane) was obtained from a set of 20–25 serial images. Significance of difference between control and autoimmune staining were estimated using the Mann-Whitney test. Staining intensity was graded in a range + (weak) to +++ (intense).

### Pre-absorption study: blockade of immunoperoxidase staining of human term placental tissue with human recombinant tTg

Serum positive for anti tTG antibodies (1:20 dilution in PBS) or mouse monoclonal anti-tTG (CUB 7204) at 1:400 dilution was incubated with recombinant human tTG (rhtTG) in different concentrations (5 μg, 2.5 μg, 1.25 μg) at 37°C for two hours prior to incubation with wax sections as described earlier.

For this experiment, mouse monoclonal anti tTG CUB 7402 (1:400 dilution) and PBS were used as positive and negative controls respectively. Goat anti mouse-HRP or rabbit anti human IgA-HRP were used in 1:40 dilutions as secondary antibodies. Controls were also carried out in which serum (1:20) or monoclonal anti-tTG were preincubated with recombinant human tTG (2.5 μg, 37°C 2 h) prior to staining. Full inhibition was achieved in both instances.

### Direct binding of IgA to the placental villus

Placental tissue was fine-dissected to produce 2–3 mm pieces with intact villous architecture including stem, intermediate and terminal branches, fixed in 4% PFA/PBS for 2 h, then washed in 1% BSA/PBS (4 × 30 min). Positive or negative serum (1:10 in 5% BSA/PBS) and mouse monoclonal anti-tTG (1:200 in 5% BSA/PBS) were used as primary antibodies. Rabbit anti-human IgA-FITC (1:40; Dako F0204) and rabbit anti-mouse IgG-FITC (1:40; Dako F0261) secondary antibodies were used. The specimens were incubated with primary and secondary antibodies, washed in PBS (×4) in foil-wrapped eppendorf tubes over 30 min on a roller-mixer, snap frozen in OCT and cryosectioned. Negative controls contained either no primary antibody or anti-β-actin antibody (1:5000 in 5% BSA/PBS). The actin antibody produced staining only in the immediate vicinity (within ~50 μm) of cut surfaces (not shown), proving that fully epithelialised placental tissue was neither permeable to immunoglobulin nor leaky.

### *In situ *tTG activity and its inhibition by autoantibody

Syncytial tTG activity was assayed *in situ *by the incorporation of a fluorescent substrate into target proteins at the surface of intact placental tissue. Tissue (2–3 mm pieces) was incubated with the acyl donor hexapeptide biotinyl-TVQQEL[28,29] (0.5 mM in serum-free F12/DMEM, 37°, 30 min). After the reaction, specimens were washed in F12/DMEM (2 × 2 min), fixed in 4% PFA (30 min), washed again (4 × 30 min), snap frozen in OCT and cryosectioned. Sections were incubated with streptavidin-FITC (1:200), washed and mounted using Vectashield containing propidium iodide. For the tTG activity inhibition assay, placental tissue pieces were directly incubated with serum (1:20 in 5% BSA/PBS) or monoclonal anti-tTG antibody (1:200) prior to addition of the acyl donor substrate.

## Results

### Autoimmune IgA binds to the placenta

The EMA tissue immunofluorescence test for CD may detect autoantibody to non-tTG targets. 132 serum samples from a general immunology clinic were therefore tested for EMA and then assayed blind for tTG antibodies (table 1). All sera positive for EMA at 1:1000 also contained anti-tTG reactivity and 60/74 (81%) of sera negative for EMA were also negative for anti-tTG. 14/74 (18.9%) of EMA-negatives were positive for anti-tTG while 13/58 (22.4%) of EMA-positives (all dilutions) were negative for the anti-tTG assay. Seven of twelve sera that were strongly positive in both assays were of sufficient volume for further experiments. Seven negative sera were selected as controls.

**Table 1:** Correlation of anti tTG immunoassay (ELISA) with EMA assays.

**OD Ratio**	**Negative for EMA**	**1 in 30** **EMA**	**1 in 100** **EMA**	**1 in 300** **EMA**	**1 in 1000** **EMA**
OD <1	60 (81.1%)	2 (33.3%)	9 (42.8%)	2 (10.5%)	0
*OD 1–1.4	2 (2.7%)	0	1 (4.8%)	1 (5.3%)	0
OD >1.4	12 (18.9%)	4 (66.6%)	11 (52.4%)	16 (84.2%)	12 (100%)

Total	74	6	21	19	12

Number of serum samples are given with and percentages in parentheses. Samples positive for EMA were tested in serial dilutions (1 in 30, 1 in 100, 1 in 300 and 1 in 1000). Anti tTG antibody results are reported as positive (OD ratio >1.4), borderline (OD ratio 1–1.4) and negative (OD ratio <1).

**Figure 1:**
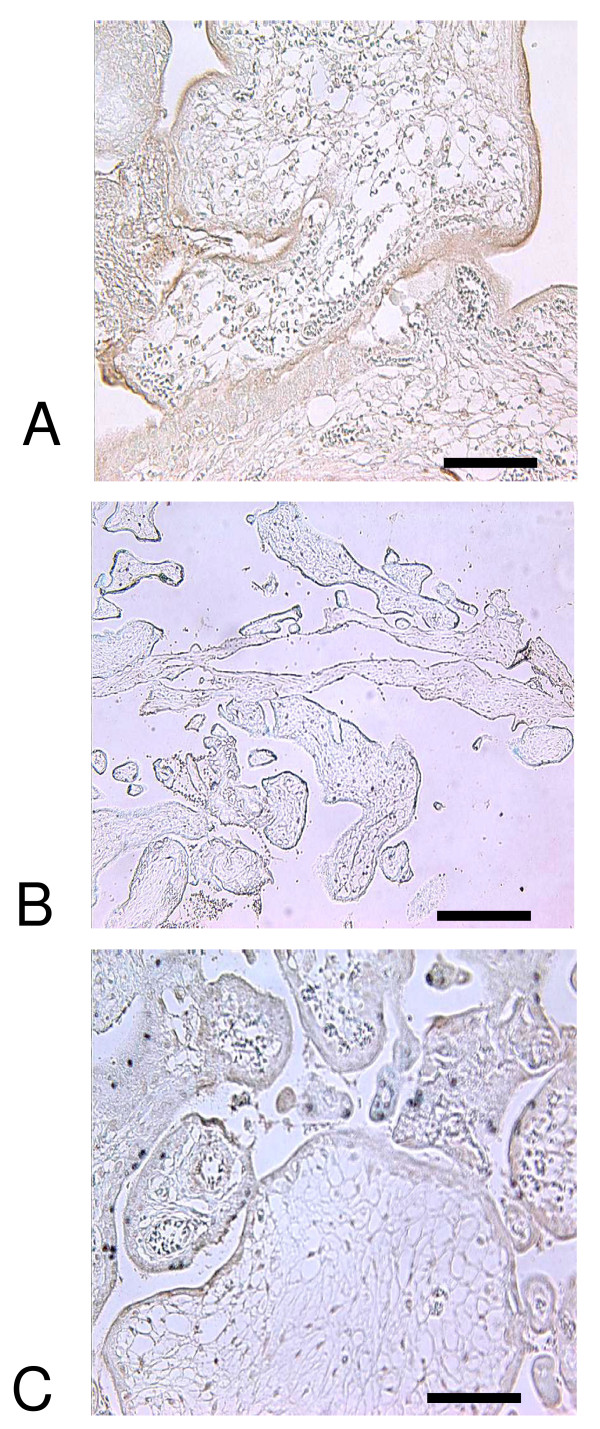
**A) Immunohistochemical staining (brown with blue hematoxylin counterstain) showing IgA from CD serum binding at the syncytial (maternal) surface of term placenta; B) binding is abolished by preincubation of serum with recombinant tissue transglutaminase; C) negative control serum fails to stain**. Scale bar: 250 μm (a, c), 60 μm (b).

Sera were used for immunohistochemistry of term placental tissue. Monoclonal anti-tTG stained strongly the syncytial microvillous membrane (MVM) as well as cytotrophoblast and stromal and vascular elements, confirming previous data (figure 1A) [26,27]. IgA could not be detected in control sections stained either with anti-human IgA secondary only (not shown) or anti-tTG-negative serum (figure 1C). All tTG-positive sera contained IgA that recognised target antigen in both cytotrophoblast and syncytial MVM (table 2) as well as endothelial and decidual cells (not shown). The intensity of staining in the MVM was variable, suggesting a non-uniform distribution over the surface of the villus (figure 1A). Antibody binding was inhibited by pre-incubation of serum with recombinant human tTG (figure 1B). Two tTG-negative sera showed weak reactivity with cytotrophoblast and syncytial MVM, while the others did not bind detectably to targets in the placenta (table 2).

**Table 2:** Immunostaining of human term placenta using anti-tTG-positive or -negative sera.

**Serum Samples**	**Anti tTG** **Positive Serum**	**Anti tTG** **Negative Serum**
Apical syncytial MVM	7/7	2/7 (the other 5 were negative)
Linear proportion stained	22–48%	15–25% **p = 0.0001
Grading of staining	++/+++	+/++
Syncytioplasm	7/7	2/7
Area stained	17–68%	15–25% **p = 0.0001
Grading of staining	+++/++	+/++

Data are given as linear proportion stained (MVM) or area stained (syncytioplasm) with an associated intensity range.

### IgA can be recruited to the placental surface from the intervilllous space

**Figure 2:**
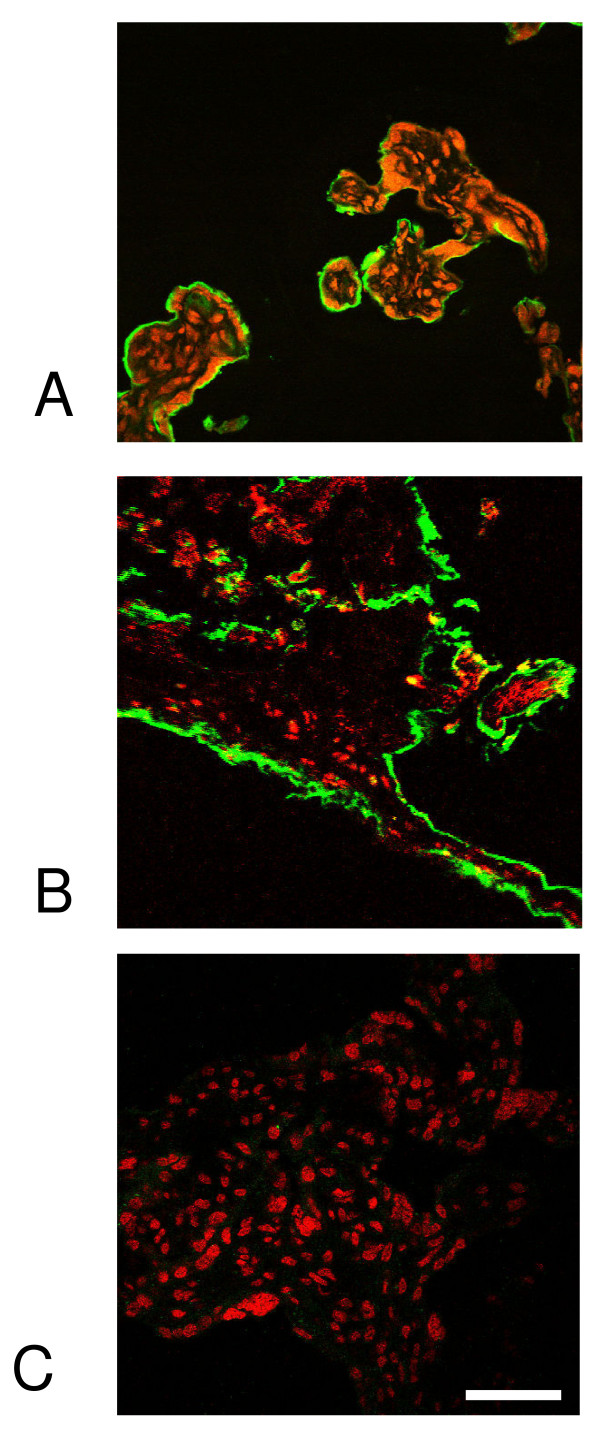
**A novel direct binding assay**. Sera positive or negative for anti-tTG antibody were directly incubated with intact placental villous tissue fragments and followed by FITC-conjugated anti-IgA. Tissue was then cryosectioned and counterstained for nuclei (PI; red). A) Positive control: mouse monoclonal anti tTG; B) serum positive for anti tTG; C) serum negative for anti-tTG. The green staining on the outer surface of the villus corresponds to syncytium. Scale bar, 100 μm.

Immunohistochemistry demonstrated binding at the MVM, but the resolution of the technique is insufficient to distinguish tTG at the outer or inner surface. A direct binding assay was developed to investigate whether maternal IgA circulating in the intervillous space could interact directly with target tTG on the outer surface of the placenta. Small pieces of villous tissue were fixed under non-permeabilising conditions, then incubated in autoimmune serum and bound IgA localised by fluorescence microscopy after sectioning. Abundant antigen could be detected at the surface of the placenta (figures 2A,B). When anti-tTG-negative serum was used, no staining was observed (figure 2C). These experiments indicate that tTG at the MVM is accessible to antibody binding from maternal circulation.

### tTG activity in the MVM is inhibited by autoantibody

**Figure 3:**
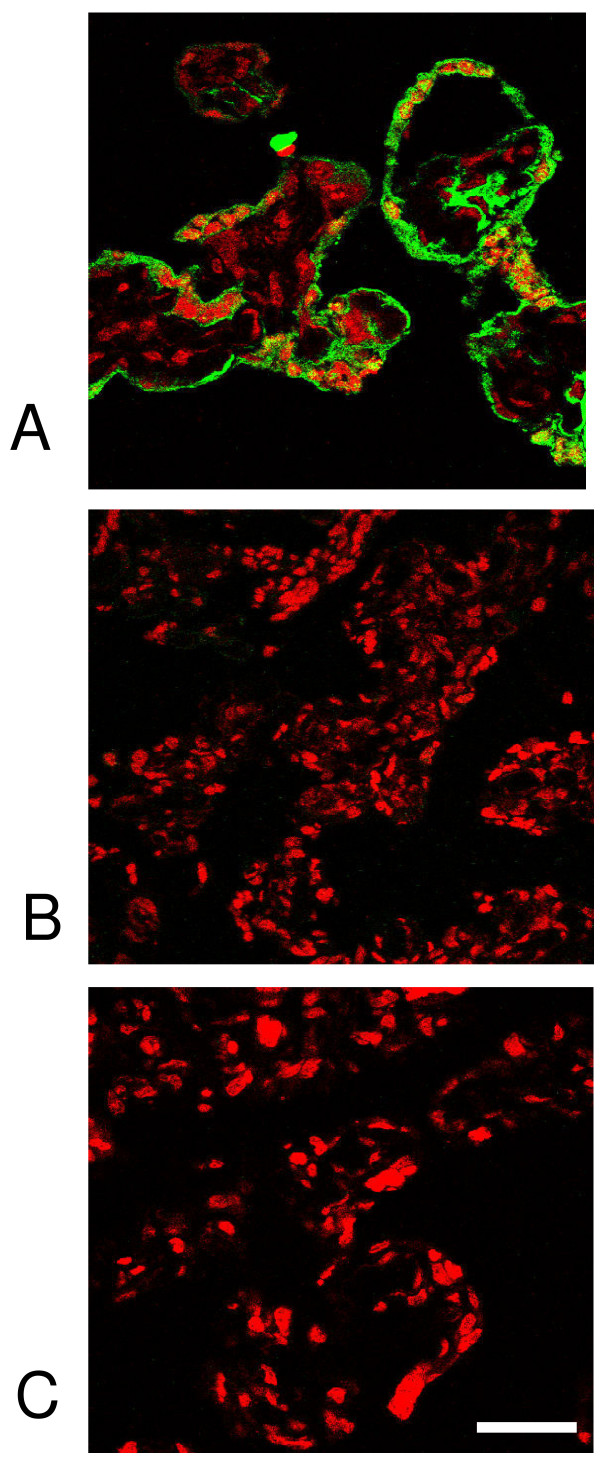
**CD serum inhibits tTG enzymatic activity at the surface of the placenta**. Villous tissue was incubated of with an acyl donor tTG substrate (biotinyl-TVQQEL), then biotin incorporation visualised by staining with a green fluorescent avidin conjugate. A) Staining in trophoblast, indicating incorporation of the substrate. This was completely abolished after tissue had been pre-treated with B) mouse monoclonal anti tTG antibody or C) positive serum. Scale bar, 100 μm.

Given the presence of accessible, immunoreactive tTG, a method was developed to detect tTG enzymatic activity in the MVM. Small pieces of villous tissue were dissected from term placenta obtained within 30 min of delivery and incubated in a biotinylated acyl donor substrate peptide. Cryosections were produced and stained with fluorescent avidin in order to reveal sites of activity. The results (figure 3) indicate substrate localised to the MVM, showing that enzyme is accessible to small substrates and is active *in situ*. When placental tissue was preincubated with monoclonal anti-tTG or with anti-tTG-positive serum before adding substrate, biotinylation was strongly inhibited (figure 3) demonstrating the ability of CD autoantibodies to alter the activity of tTG in the placental MVM.

## Discussion

In previous studies we used immunohistochemistry of placental tissue sections to establish that tTG is widely expressed, and biochemical assays to show active tTG in the MVM[26,28]. The results now reported show that CD sera contain IgA that binds to tTG in the same placental sites. However the placenta is an effective immunological barrier, and in order to establish in principle that placental autoantigen may be accessible to the maternal immune system, it was necessary to devise a direct binding assay using intact villous tissue. The data obtained prove unequivocally that placental tTG is accessible to binding of maternal autoantibody, suggesting that it is associated with the outer face of the MVM. Using *in situ *enzymology we have shown that the enzyme is active at this site and is inhibited both by monospecific anti-tTG antibody and by CD sera.

The results indicate the likelihood that placental tTG activity is impaired in CD pregnancies. The consequences for function are not yet clear but as the major site of nutrient exchange, the MVM is vulnerable to pathogenic effects that may impair membrane-associated processes including nutrient import. Altered apoptosis has been reported in CD placentas[30]. We have postulated [28] that tTG plays a role in stabilizing fragments shed from the syncytiotrophoblast microvillous membrane into maternal circulation. Particle cross-linking may stabilise the constituent supramolecular complexes which may therefore be more efficiently phagocytosed. Inhibition of cross-linking by autoantibody may cause destabilization of the particulate fraction with consequent release of soluble fetoplacental antigen and exposure of the maternal immune system to an altered immunological challenge from the hemiallogeneic placenta. In turn this may have wider systemic consequences for immune recognition of the conceptus. These ideas will require experimental validation, but in the meantime the data presented add support to the case for screening for CD in early pregnancy with the intent to offer a gluten-free diet to anti-tTG antibody-positive women.

## Abbreviations

(OD): Absorbance; (anti tTG antibody): Anti tissue transglutaminase antibody; (EMA): Endomysium antibodies; (Av PO): Avidin peroxidase; (CD): Coeliac disease; (DAB): 3, 3' diaminobenzidine tetrahydrochloride dehydrate; (FITC): Fluorescein isothiocyanate; (GFD): Gluten-free diet; (HRP): Horseradish peroxidase; (rhtTG): Human recombinant tissue transglutaminase; (IgA): Immunoglobulin A; (IgG): Immunoglobulin G; (IUGR): Intrauterine growth restriction; (MVM): Microvillous membrane; (PFA): Paraformaldehyde; (PBS): Phosphate buffered saline; (PI): Propidium Iodide; (tTG): Tissue transglutaminase; (TBS): Tris-buffered Saline.

## Competing interests

The authors declare that they have no competing interests.

## Authors' contributions

JDA PNB and NA designed the study. NA carried out the experiments. NJR contributed to assay development. NA and JDA analysed the data. JDA wrote the paper which was read and approved by the other authors.
